# Unmet foot health needs in people with Systemic Sclerosis: an audit of foot health care provision

**DOI:** 10.1186/1757-1146-3-S1-O2

**Published:** 2010-12-20

**Authors:** Begonya Alcacer-Pitarch, Heidi Siddle, Mathew Christie, Maya Buch, Paul Emery, Anthony Redmon

**Affiliations:** 1 Section of Musculoskeletal Disease, University of Leeds, Leeds, UK

## Introduction

Systemic Sclerosis (SSc) has a potentially disabling effect on the foot. The UK PRCA/ARMA standards of care indicate that all people with SSc should receive at least basic information about their foot health, and those with foot problems should have access to self-management advice and care where needed. The aim of this audit was to compare the concordance of foot health services offered in Leeds (UK) for people who have SSc, with nationally agreed standards of care.

## Methods

This audit comprised 91 patients with SSc who were attending either the connective tissue disease outpatient clinic (n=70) or the specialist rheumatology foot health clinic at Chapel Allerton Hospital (n=21). All the patients completed a disease-specific audit tool developed by the UK PRCA.

## Results

Of the 91 patients, 78 were female and 10 were male (3 unknown) with a mean age of 58 years (range 20-83). In our sample only 35(39%) out of 91 patients reported having received information about their foot health. Fifty-five (60%) of 91 reported having had foot problems in the past, with 54 (59%) reporting current foot problems. Forty-nine (54%) out of 91 reported having had access to foot care previously and 38 (42%) were receiving foot health care currently. Of the 55 patients that had foot problems, a slightly higher proportion 32 (58%) had received foot health information. Forty-five(82%) of the 55 patients with foot problems, reported previous access to foot health care provided through a variety of services: 29 (53%) in a hospital based specialist rheumatology foot health clinic; 5 (9%) in community care podiatry department; 4 (7%) in private podiatry practice; 4 (7%) in general practice; 3 (6%) others. The 21 patients attending the foot health clinic received the following interventions: 21(100%) received toe nail care; 7(33%) finger nail care; 20 (95%) corn and callus reduction; 11(52%) insole provision. The profile of specialist care was: 16 (76%) receiving ulcer care; 3 (14%) nail surgery; with 1 (5%) referred for orthopaedic opinion; and 2 (10%) for specialist vascular assessment.

## Discussion

This audit demonstrates that patients with SSc have a relatively high prevalence of foot problems, however this study also shows that access to foot health services is limited. The PRCA/ARMA standards of care recommend that all people with SSc should receive at least basic information about their foot health but this is not reflected in this study.

